# Supplementary material for: Global hypo-methylation in a proportion of glioblastoma enriched for an astrocytic signature is associated with increased invasion and altered immune landscape
Source: eLife. 2022 Nov 22;11:e77335. doi: 10.7554/eLife.77335 (PMC9681209; doi:10.7554/eLife.77335)
Supplement: Figure 2—source data 1. [file elife-77335-fig2-data1.zip › Figure_2_source_data_1/Figure_2B/homerResults/motif11.similar.html]

motif11

## Information for motif11

A
T
G
C
A
T
C
G
A
T
G
C
G
T
A
C
G
C
A
T
T
A
C
G
T
A
G
C
C
G
T
A
A
T
C
G
A
T
G
C
A
G
T
C
G
A
T
C
G
C
A
T
A
C
T
G
A
T
C
G
  
Reverse Opposite:  

A
T
G
C
T
G
A
C
C
G
T
A
C
T
A
G
T
C
A
G
T
A
C
G
T
A
G
C
G
C
A
T
A
T
C
G
A
T
G
C
C
G
T
A
C
A
T
G
T
A
C
G
T
A
G
C
T
A
C
G
  

|  |  |
| --- | --- |
| p-value: | 1e-67 |
| log p-value: | -1.556e+02 |
| Information Content per bp: | 1.853 |
| Number of Target Sequences with motif | 116.0 |
| Percentage of Target Sequences with motif | 5.02% |
| Number of Background Sequences with motif | 2.5 |
| Percentage of Background Sequences with motif | 0.71% |
| Average Position of motif in Targets | 92.0 +/- 54.0bp |
| Average Position of motif in Background | 103.7 +/- 52.3bp |
| Strand Bias (log2 ratio + to - strand density) | 0.3 |
| Multiplicity (# of sites on avg that occur together) | 1.05 |
| Motif File: | file (matrix) reverse opposite |

### Similar de novo motifs found

|  |  |  |  |  |  |  |  |
| --- | --- | --- | --- | --- | --- | --- | --- |
| Rank | Match Score | Redundant Motif | P-value | log P-value | % of Targets | % of Background | Motif file |
| 1 | 0.615 | A C G T A G T C A C G T A C T G A C T G A C T G A G T C A C G T A C T G A G T C | 1e-40 | -93.105440 | 3.72% | 0.80% | motif file (matrix) |
| 2 | 0.612 | A G T C A C G T A C T G A G T C C G T A A C T G A G T C C G T A A G T C A G T C | 1e-32 | -74.505924 | 3.29% | 0.77% | motif file (matrix) |
| 3 | 0.615 | C G T A A C T G A C T G A C T G A G T C C G T A A C T G A G T C A C T G A C T G | 1e-27 | -63.982053 | 3.03% | 0.85% | motif file (matrix) |
| 4 | 0.614 | A G T C A C G T A C T G A C T G A C T G A G T C A C G T A C G T A G T C C G T A A C T G A G T C | 1e-20 | -47.212249 | 3.20% | 0.87% | motif file (matrix) |
